# Advancements in Robotic Surgery: A Comprehensive Overview of Current Utilizations and Upcoming Frontiers

**DOI:** 10.7759/cureus.50415

**Published:** 2023-12-12

**Authors:** Kavyanjali Reddy, Pankaj Gharde, Harshal Tayade, Mihir Patil, Lucky Srivani Reddy, Dheeraj Surya

**Affiliations:** 1 Surgery, Jawaharlal Nehru Medical College, Datta Meghe Institute of Higher Education and Research, Wardha, IND; 2 Obstetrics and Gynaecology, Jawaharlal Nehru Medical College, Datta Meghe Institute of Higher Education and Research, Wardha, IND

**Keywords:** healthcare innovation, surgical advancements, medical technology, surgical robotics, minimally invasive surgery, robotic surgery

## Abstract

Robotic surgery, a groundbreaking advancement in medical technology, has redefined the landscape of surgical procedures. This comprehensive overview explores the multifaceted world of robotic surgery, encompassing its definition, historical development, current applications, clinical outcomes, benefits, emerging frontiers, challenges, and future implications. We delve into the fundamentals of robotic surgical systems, examining their components and advantages. From general and gynecological surgery to urology, cardiac surgery, orthopedics, and beyond, we highlight the diverse specialties where robotic surgery is making a significant impact. The many benefits discussed include improved patient outcomes, reduced complications, faster recovery times, cost-effectiveness, and enhanced surgeon experiences. The outlook reveals a healthcare landscape where robotic surgery is increasingly vital, enabling personalized medicine, bridging healthcare disparities, and advancing surgical precision. However, challenges such as cost, surgeon training, technical issues, ethical considerations, and patient acceptance remain relevant. In conclusion, robotic surgery is poised to continue shaping the future of health care, offering transformative possibilities while emphasizing the importance of collaboration, innovation, and ethical governance.

## Introduction and background

Robotic surgery, a cutting-edge field within the broader realm of medical technology, has transformed the landscape of surgical procedures. Combining robotics's precision with human surgeons' expertise has opened up new horizons in medical practice. This review article delves into the multifaceted domain of robotic surgery, offering a comprehensive overview of its current utilizations and upcoming frontiers [1,2].

Robotic surgery, also known as robot-assisted surgery, refers to a minimally invasive surgical technique where specialized robotic systems are employed to assist surgeons in performing procedures with unparalleled precision and control. These systems consist of robotic arms equipped with surgical instruments, a surgical console operated by the surgeon, and a high-definition vision system that provides a magnified 3D view of the surgical site. Robotic surgery is distinct from traditional open surgery and conventional laparoscopy, as it combines the surgeon's skills with robotic technology to enhance the quality and safety of procedures [3].

The roots of robotic surgery trace back to the mid-20th century when the idea of using machines to aid in surgery first emerged. However, it was in the late 20th century that significant progress was made. In 1985, the PUMA 560, a pioneering robotic system, performed neurosurgical biopsies. This marked the beginning of robotic surgery as we know it today [4].

The watershed moment in the history of robotic surgery occurred with the introduction of the da Vinci Surgical System in the early 2000s. Developed by Intuitive Surgical, this system revolutionized minimally invasive surgery by providing surgeons with enhanced dexterity, precision, and improved visualization. The da Vinci Surgical System quickly gained acceptance in various surgical specialties, paving the way for the rapid expansion of robotic-assisted procedures [5].

The primary purpose of this overview is to provide a comprehensive and up-to-date exploration of the world of robotic surgery. It seeks to inform healthcare professionals, researchers, policymakers, and the general public about the current state of robotic surgery, its applications, and the emerging frontiers that hold promise for the future. By examining the advancements and challenges in this field, this article aims to contribute to a broader understanding of the role of robotic surgery in modern medicine.

The significance of advancements in robotic surgery cannot be overstated. These developments have improved patient outcomes, reduced complications, shorter hospital stays, and enhanced surgical precision. Moreover, they have opened doors to new possibilities, such as telesurgery, personalized medicine, and the integration of artificial intelligence (AI). The potential to democratize surgical expertise, improve access to health care, and redefine the boundaries of what is achievable in surgery underscores the importance of staying abreast of the latest robotic surgical innovations. This overview serves as a critical resource for comprehending the evolving landscape of robotic surgery and its profound impact on health care [6].

## Review

Fundamentals of robotic surgery

Robotic Surgical Systems

Da Vinci surgical system:* *The Da Vinci system features multiple robotic arms with surgical instruments. These arms precisely mimic the surgeon's hand movements and provide a more excellent range of motion than traditional laparoscopic instruments [7]. The surgeons control the robotic arms from a surgical console in the operating room. The console offers a 3D view of the surgical field and hand controls that allow the surgeon to manipulate the instruments precisely [8]. High-definition cameras on the robotic arms provide a detailed view of the surgical site. The vision system offers 3D visualization and magnification, enhancing the surgeon's ability to navigate complex anatomical structures [9]. The Da Vinci system has been employed across various surgical specialties, including urology, gynecology, general surgery, and cardiac surgery. Its success is attributed to its ability to enhance surgical precision, reduce invasiveness, and expedite patient recovery.

Competing Systems

While the Da Vinci system remains dominant, several emerging competitors and alternative robotic surgical systems have entered the field. Medtronic, Stryker, and Titan Medical have developed robotic platforms to provide alternative solutions for minimally invasive surgery [10]. These competing systems offer healthcare providers and patients additional choices for robotic-assisted procedures, fostering innovation and competition within robotic surgery. These alternatives contribute to robotic surgical technology's ongoing development and advancement [1].

Key Components and Features

Robotic arms: They are the mechanical extensions of robotic surgical systems that replicate the movements of the surgeon's hands with remarkable precision. These arms are equipped with surgical instruments and can access tight spaces within the body, making them essential for intricate procedures. The articulation and dexterity of robotic arms enable surgeons to manipulate tissues and perform tasks accurately [11].

Surgical console: It is the command center where the surgeon sits to control the robotic arms during the procedure. It includes hand controls and foot pedals that allow the surgeon to manipulate the instruments with finesse and accuracy. The robotic arms within the patient's body translate the surgeon's movements into precise actions. The console also provides a 3D view of the surgical site through high-definition screens, enhancing visualization and enabling the surgeon to make informed decisions during the procedure [3].

Vision system: It is a critical component of robotic surgical systems, offering high-definition 3D visualization of the surgical field. This system gives surgeons a magnified, detailed view of the operative site, which is essential for performing delicate maneuvers and precise incisions. The clarity of visual feedback enables surgeons to navigate complex anatomical structures, confidently enhancing surgical precision [12].

Instruments and Tools

Robotic surgical instruments are specially designed to replicate the human hand's range of motion while filtering out any tremors or unintended movements. These instruments are vital for performing various surgical tasks, including suturing, dissecting, cauterizing, and cutting. They are interchangeable during procedures, allowing for versatility and adaptability to the specific requirements of each surgery. The advanced instrumentation contributes to robotic surgery's overall precision and effectiveness [13].

Advantages of Robotic Surgery

Precision: Robotic systems excel in precision, enabling surgeons to perform intricate and delicate maneuvers with accuracy that surpasses what the human hand alone can achieve. The robotic arms' stability and precision reduce the risk of errors during surgery, leading to improved patient outcomes and reduced postoperative complications [14].

Enhanced dexterity: Robotic surgical systems are equipped with robotic arms that can rotate 360 degrees and mimic the natural movements of a surgeon's hand but with significantly reduced tremors. This enhanced dexterity is particularly valuable for working in confined spaces within the body and efficiently performing complex tasks. Surgeons can manipulate tissues and instruments with high control, enhancing their surgical capabilities [15].

Three-dimensional visualization: Robotic systems provide high-quality 3D visualization of the surgical field, offering depth perception and spatial awareness that surpasses traditional 2D laparoscopy. This enhanced visual feedback greatly benefits surgeons by improving their ability to navigate anatomical structures safely and accurately. It allows for better identification of critical structures and precise instrument placement [16].

Reduced surgeon fatigue: Robotic surgery minimizes the physical strain on surgeons. Unlike traditional open surgery, which often requires prolonged standing and holding heavy instruments, robotic surgery allows surgeons to operate comfortably from a seated position at the surgical console. This reduction in physical fatigue enables surgeons to maintain peak performance throughout lengthy procedures, ultimately enhancing patient safety and surgical outcomes [17].

Current utilization of robotic surgery

General Surgery

Cholecystectomy: Robotic-assisted cholecystectomy, the gallbladder removal, is one of the most common general surgical procedures performed robotically. Robotic systems provide surgeons with enhanced precision, which is particularly important when working in the confined space of the abdomen. The robotic instruments reduce the risk of injury to surrounding structures, such as the bile ducts and blood vessels. Patients undergoing robotic cholecystectomy benefit from smaller incisions, reduced postoperative pain, and faster recovery [18].

Hernia repair: Robotic-assisted hernia repair has become increasingly popular due to its precision and minimally invasive techniques. Robotic systems allow surgeons to perform precise mesh placement for hernia repair, reducing the risk of recurrence. Smaller incisions, less postoperative pain, and faster recovery times make robotic hernia repair an attractive option for patients seeking a quicker return to their daily activities [19].

Appendectomy: The removal of the appendix, known as an appendectomy, can be accomplished robotically. Robotic appendectomy involves smaller incisions and precise instrumentation, reducing tissue trauma and postoperative pain. Patients benefit from quicker recovery times and a shorter hospital stay than traditional open appendectomy [20].

Gynecological Surgery

Hysterectomy: Robotic-assisted hysterectomy has gained popularity for treating various gynecological conditions, including fibroids and endometriosis. During a robotic hysterectomy, the uterus is removed with robotic instruments, resulting in smaller incisions and reduced scarring. This minimally invasive approach offers several advantages, including shorter hospital stays, quicker recovery times, and improved cosmetic outcomes. Patients undergoing robotic hysterectomy often experience less postoperative pain and a faster return to normal activities [21].

Ovarian cystectomy: Removing ovarian cysts can be performed robotically with enhanced precision. Robotic systems allow surgeons to navigate delicate ovarian tissues with dexterity, minimizing the risk of damage and reducing blood loss during the procedure. Patients benefit from smaller incisions, less postoperative discomfort, and faster recovery times. Robotic ovarian cystectomy is particularly valuable for preserving ovarian function in cases where cyst removal is required [22].

Myomectomy: Surgical removal of uterine fibroids, known as myomectomy, can be performed robotically. This approach allows surgeons to target and remove fibroids while preserving the uterus, making it an option for women who wish to retain their fertility. Robotic myomectomy offers improved precision and control, reducing tissue damage and a faster patient return to normal activities. The minimally invasive procedure also leads to less scarring and postoperative pain [23].

Urological Surgery

Prostatectomy: Robotic prostatectomy is a widely adopted treatment for prostate cancer. This procedure involves the removal of the prostate gland. Robotic systems give surgeons enhanced precision and dexterity, allowing them to spare nerves critical for urinary continence and sexual function. Smaller incisions and improved visualization contribute to reduced postoperative pain and shorter hospital stays. Patients undergoing robotic prostatectomy experience faster recovery times and improved quality of life compared to traditional open surgery [24].

Nephrectomy: Partial or complete kidney removal due to tumors, kidney disease, or donation can be performed robotically. Robotic nephrectomy offers the advantage of smaller incisions, which result in less pain, reduced scarring, and a quicker return to normal activities. Patients also benefit from shorter hospital stays and a lower risk of postoperative complications, making robotic nephrectomy a preferred option for many [25].

Pyeloplasty: Robotic pyeloplasty is a highly effective treatment for ureteropelvic junction (UPJ) obstruction, which causes blockage in the urinary system. Robotic systems assist surgeons in precisely reconstructing the UPJ, ensuring proper urine flow. This minimally invasive approach results in shorter recovery times and high success rates for resolving the obstruction. Patients can expect improved renal function and symptom relief following the procedure [26].

Cardiac Surgery

Coronary artery bypass grafting (CABG): Robotic-assisted CABG has transformed the landscape of cardiac surgery. This technique allows for the precise revascularization of blocked coronary arteries with smaller incisions than traditional open-heart surgery. Surgeons use robotic systems to access the heart through small ports, reducing trauma to the chest wall. The robotic arms provide a high degree of dexterity and accuracy for suturing and grafting, resulting in improved outcomes, reduced postoperative pain, and faster patient recovery [27].

Mitral valve repair/replacement: Robotic systems have greatly improved the precision and effectiveness of mitral valve repair or replacement surgeries. Surgeons can perform these intricate procedures with the assistance of robotic arms, which provide enhanced dexterity and control. Robotic-assisted mitral valve surgery is associated with smaller incisions, reduced blood loss, and shorter hospital stays. Patients benefit from improved valve function and minimized chest trauma, resulting in a faster return to normal activities [28].

Atrial fibrillation ablation: Robotic systems are increasingly used in minimally invasive procedures for treating atrial fibrillation, a common heart rhythm disorder. Surgeons use robotic instruments to access the heart's atria, performing ablation procedures to restore normal rhythm. Robotic-assisted atrial fibrillation ablation offers precise lesion creation and mapping, reducing the risk of complications and the need for repeat procedures. Patients experience shorter recovery times and a lower risk of postoperative complications [29].

Orthopedic Surgery

Total knee arthroplasty (TKA): Robotic-assisted TKA has revolutionized knee replacement surgery. It enhances the alignment and positioning of knee implants with high precision. Surgeons use robotic systems to create a personalized surgical plan based on the patient's anatomy. During the procedure, the robot assists the surgeon in executing the plan, ensuring accurate implant placement. This precision improves long-term outcomes, reduces implant wear and tear, and enhances patient satisfaction [30].

Total hip arthroplasty (THA): Robotic systems have also improved the precision and accuracy of hip replacement surgery. THA procedures benefit from robotic technology, which allows for meticulous preoperative planning and intraoperative guidance. The robot assists the surgeon in optimizing implant placement, reducing the risk of complications such as dislocation and leg length discrepancy. Patients undergoing robotic-assisted THA experience improved joint function and a lower likelihood of revision surgeries [31].

Spinal surgery: Robotic technology is increasingly used in spinal surgery to enhance precision and minimize the invasiveness of procedures. Some spinal surgeries, such as spinal fusion, are performed robotically. Robotic systems assist surgeons in creating detailed maps of the spine and guide the placement of implants and instrumentation. The result is smaller incisions, reduced tissue damage, and greater accuracy in achieving spinal stability. Patients benefit from improved postoperative pain management and faster recovery [32].

Head and Neck Surgery

Transoral robotic surgery (TORS): Transoral robotic surgery treats head and neck cancers, allowing for the precise removal of tumors while minimizing trauma to surrounding tissues. TORS offers several advantages, including improved access to difficult-to-reach areas, reduced postoperative pain, and quicker recovery times. It is particularly beneficial for patients with oropharyngeal cancers and offers the potential for improved speech and swallowing outcomes [33].

Thyroidectomy: Robotic-assisted thyroid surgery is performed through small incisions, offering a minimally invasive approach for thyroid gland removal. This approach minimizes scarring and improves cosmetic outcomes for patients. Robotic thyroidectomy is often employed in thyroid nodules, thyroid cancer, and hyperthyroidism, providing surgeons with enhanced visualization and precision during the procedure [34].

Parathyroidectomy: The removal of overactive parathyroid glands can be performed robotically with high precision. Robotic parathyroidectomy offers advantages such as smaller incisions, reduced postoperative discomfort, and shorter recovery times. The precision of robotic instruments allows surgeons to target the affected glands accurately, improving patient outcomes and reducing the risk of complications associated with hyperparathyroidism [34].

Clinical outcomes and benefits

Improved Patient Outcomes

Enhanced precision: Robotic systems provide unparalleled precision in surgical procedures. Surgeons can perform intricate tasks with submillimeter accuracy, minimizing tissue damage and optimizing the surgical outcome. The ability to precisely control robotic instruments benefits patients by reducing the risk of complications and postoperative issues [3].

Reduced blood loss: The precise control offered by robotic instruments can lead to significantly reduced blood loss during surgery. This reduction in blood loss is critical in minimizing the need for blood transfusions and the associated risks, contributing to safer and more successful surgical outcomes [35].

Smaller incisions: Minimally invasive robotic procedures typically require smaller incisions than open surgery. These smaller incisions result in several patient benefits, including less pain, reduced scarring, and a lower risk of infection. Patients appreciate the cosmetic advantages of smaller incisions and experience less postoperative discomfort [33].

Shorter hospital stays: Robotic surgery often leads to faster recovery and reduced postoperative complications. Many patients can be discharged from the hospital sooner, enhancing their overall quality of life and reducing healthcare costs associated with prolonged hospitalization [36].

Lower risk of infection: Minimally invasive robotic procedures carry a lower risk of surgical site infections due to smaller incisions and reduced exposure to external contaminants. Lower infection rates contribute to improved patient safety and satisfaction [37].

Reduced Complications

Lower infection rates: Robotic surgery's minimally invasive approach involves smaller incisions and reduced handling of tissues, which minimizes the risk of postoperative infections. Smaller incisions result in less exposure of internal tissues to the external environment, reducing the likelihood of contamination. Lower infection rates are particularly significant in complex surgeries where infection can lead to severe complications [36].

Fewer complications: The precision of robotic instruments and enhanced visualization provided by high-definition 3D imaging contribute to fewer intraoperative and postoperative complications. Surgeons can manipulate tissues with high accuracy and control, reducing the risk of unintended damage or bleeding. Improved visualization allows for meticulous surgical techniques, minimizing the risk of complications during the procedure and improving patient safety [38].

Reduced postoperative pain: Robotic surgery's smaller incisions and minimized tissue trauma often lead to less postoperative pain than open surgery. Patients experience less discomfort, which reduces the need for pain medication and promotes faster recovery. This enhanced postoperative comfort improves patient experience and satisfaction [39].

Shorter hospital readmissions: Patients who undergo robotic surgery are less likely to require readmission to the hospital for postoperative complications. The reduced risk of complications and infections, coupled with faster recovery times, means that patients can often return home sooner and with greater confidence in their recovery [40].

Faster Recovery Times

Quicker return to normal activities: Robotic surgery's minimally invasive approach offers the advantages of reduced pain, smaller incisions, and fewer complications than traditional surgery. As a result, patients can typically resume their daily routines and activities more rapidly. Whether returning to work, caring for their families, or engaging in physical activities, patients experience less disruption. This accelerated return to normalcy improves the quality of life and patient satisfaction [28].

Shorter hospital stays: Many robotic surgical procedures are performed outpatient or short-stay. Patients can often go home on the same day or within a short period after surgery. This minimizes the disruption to a patient's life and reduces healthcare costs associated with more extended hospitalizations. Shorter hospital stays translate to more efficient use of healthcare resources, freeing up beds and medical staff for other needy patients [28].

Less postoperative fatigue: Robotic surgery reduces postoperative fatigue and discomfort. Patients report less pain and physical strain after minimally invasive procedures, which enables a faster return to their usual level of energy and mobility. This reduction in postoperative fatigue is particularly significant for elderly or frail patients, as it allows them to recover with less physical and emotional stress [28].

Cost-Effectiveness

Reduced hospitalization costs: Robotic surgery often leads to shorter hospital stays than traditional open surgery, reducing overall hospitalization costs. Patients who undergo minimally invasive robotic procedures typically experience less pain, reduced risk of infection, and faster recovery times. These factors contribute to shorter postoperative hospitalization, freeing healthcare resources and reducing associated costs. Decreased stay also enhances patient comfort and satisfaction [41].

Faster recovery means an earlier return to work: The faster recovery associated with robotic surgery benefits patients by allowing them to return to work and normal activities sooner. This reduces financial strain for individuals due to fewer days off work and a quicker return to their productive roles. Additionally, shorter recovery times can decrease the need for temporary disability benefits, reducing indirect costs related to lost productivity [41].

Lower complication rates: Robotic surgery has been shown to have lower complication rates compared to traditional surgical approaches. Fewer complications mean reduced healthcare costs associated with follow-up surgeries, treatments, and hospital readmissions. Avoiding complications also contributes to improved patient outcomes and quality of life, which can lead to further cost savings over the long term [42].

Long-term benefits: The long-term benefits of robotic surgery go beyond immediate cost reductions. Improved patient outcomes and lower postoperative complications can result in ongoing cost savings by avoiding expensive healthcare interventions, such as prolonged hospitalizations, additional surgeries, and extensive rehabilitation. These long-term benefits contribute to the overall cost-effectiveness of robotic surgery programs [6].

Surgeon Experience and Training

Enhanced surgical skills: Robotic systems allow surgeons to enhance their surgical skills and perform complex procedures with greater precision. Robotic instruments' intuitive interfaces and dexterity allow surgeons to refine their techniques and tackle challenging cases more effectively. The high-definition 3D visualization and fine instrument control improve surgical outcomes, ultimately benefiting patients by reducing the risk of complications and postoperative issues. Continuous practice and experience with robotic surgery empower surgeons to provide high-quality, minimally invasive care across various specialties [3].

Reduced physical strain: Robotic surgery minimizes the physical strain experienced by surgeons during procedures. Unlike traditional surgery, where surgeons often maintain physically demanding positions for extended periods, robotic surgeons operate from a seated position at the console. This ergonomic advantage reduces the risk of musculoskeletal injuries and fatigue, contributing to the long-term well-being of surgical teams. Surgeons can perform intricate procedures more comfortably and precisely, enhancing their overall job satisfaction and longevity [43].

Structured training programs: To ensure safety and proficiency, comprehensive training programs are readily available for surgeons interested in adopting robotic surgical techniques. These structured programs offer hands-on training, simulation-based exercises, and mentorship opportunities. Surgeons in training can gain the necessary skills and knowledge to operate robotic systems effectively and safely. Training programs also emphasize patient safety and ethical considerations, ensuring that surgeons are well prepared to provide their patients with the highest quality of care. As robotic surgery becomes more widespread, the availability of structured training programs helps build a skilled and competent workforce of robotic surgeons [44].

Emerging frontiers in robotic surgery

Miniaturization of Robotic Systems

Micro-robotics: Advancements in miniaturization have given rise to micro-robotics, tiny robotic devices designed to perform targeted tasks within the human body. These miniature robots hold significant promise for various applications, including precise drug delivery, tissue repair, and even exploratory surgery in hard-to-reach areas. Micro-robots can navigate through intricate anatomical structures with high precision, enabling minimally invasive interventions that reduce trauma to surrounding tissues. Surgeons can remotely control them to carry out delicate procedures or deliver therapeutic agents directly to specific sites, offering new possibilities for personalized medicine and minimally invasive surgery [45].

Single-port robotic surgery: Miniaturized robotic systems have paved the way for single-port robotic surgery, a minimally invasive approach in which multiple robotic instruments are inserted through a single incision. This technique reduces the invasiveness of surgery, resulting in smaller scars, reduced postoperative pain, and faster recovery times for patients. Single-port robotic surgery leverages miniaturized robots' compact design and dexterity to perform various procedures, from gynecological and urological surgeries to gastrointestinal interventions. By consolidating multiple instruments into a single entry point, surgeons can provide patients with the benefits of minimally invasive surgery while optimizing cosmetic outcomes and minimizing the risk of complications [46].

AI and Machine Learning (ML) Integration

Autonomous robotic surgery: AI and ML algorithms are at the forefront of enabling autonomous robotic surgery. These systems can analyze vast amounts of patient data, assist with surgical planning, and sometimes perform certain aspects of surgery with minimal human intervention. Autonomous robotic surgery is a groundbreaking development that holds the potential to enhance the precision and efficiency of procedures. By relying on AI-driven automation, surgeons can benefit from real-time data analysis, assistance in decision-making, and the ability to perform tasks with submillimeter precision. While full autonomy is a long-term goal, current implementations involve a collaborative approach, where robots and surgeons work together to optimize surgical outcomes [47].

Predictive analytics: AI algorithms are increasingly utilized to predict surgical outcomes and identify potential complications. These algorithms can provide surgeons valuable insights during procedures by analyzing patient data, including medical history, imaging, and real-time surgical data. Predictive analytics enable surgeons to anticipate challenges and make real-time adjustments, ultimately improving patient safety and surgical outcomes. Surgeons can receive alerts or recommendations from AI systems that highlight critical factors to consider during surgery, such as the risk of bleeding, tissue damage, or postoperative complications. This proactive approach enhances the surgeon's decision-making process and contributes to more successful procedures [48].

Telesurgery and Remote Surgery

Telesurgery: Telesurgery, also known as remote surgery, leverages robotic systems and high-speed internet connections to enable surgeons to perform procedures on patients in different locations. This transformative technology brings specialized surgical expertise to underserved or remote areas, addressing healthcare disparities and expanding access to advanced surgical care. Telesurgery is particularly valuable in responding to emergencies, as it allows remote surgeons to provide critical surgical interventions quickly and efficiently. Robotic systems' real-time control and precision ensure that patients receive high-quality care, even when distance separates them from the surgical team [49].

Training and education: Telesurgery is crucial in surgical training and education. It allows experienced surgeons to mentor and guide less-experienced colleagues in real time, fostering skill development and knowledge transfer. Surgeons in training can benefit from remote guidance during procedures, receiving valuable feedback and guidance from mentors elsewhere. This approach enhances the proficiency of surgical teams, particularly in regions where access to comprehensive training programs may be limited. Additionally, telesurgical platforms facilitate collaborative learning and peer-to-peer knowledge sharing among surgeons, promoting continuous improvement in surgical techniques and patient care [50].

Nanorobots in Surgery

Nanomedicine: Nanorobots, operating at the nanoscale, are at the forefront of research in nanomedicine. These minuscule machines have the potential to revolutionize health care by enabling targeted drug delivery, early cancer detection, and cellular-level tissue repair. Nanorobots can be engineered to carry payloads of therapeutic agents directly to diseased or damaged cells, sparing healthy tissue. This precision in drug delivery reduces side effects and enhances the effectiveness of treatments. Additionally, nanorobots with sensors can detect disease-associated biomarkers, enabling early diagnosis and intervention. The intersection of nanorobots and surgery promises a future where diseases can be treated at their earliest stages, with minimal invasiveness and maximal efficacy [51].

Intravascular nanorobots: Intravascular nanorobots represent a groundbreaking application of nanotechnology in surgery. These microscopic robots have the potential to navigate the bloodstream with remarkable precision. They could be employed to remove blockages in blood vessels, repair damaged vascular tissues, or deliver medications directly to target sites. Intravascular nanorobots can access locations challenging for traditional surgical tools to reach, offering a minimally invasive approach to treating cardiovascular and cerebrovascular diseases. Their ability to perform intricate tasks within the circulatory system can improve patient outcomes by reducing the risks associated with conventional surgical interventions [52].

Haptic Feedback and Sensory Augmentation

Haptic feedback: Advances in haptic technology have introduced the capability of providing surgeons with tactile feedback during robotic procedures. This haptic feedback enables surgeons to "feel" the resistance and texture of tissues as they manipulate robotic instruments. By simulating the sensation of touch, surgeons gain a more intuitive understanding of the surgical environment, enhancing their control and precision. This sensory input is invaluable in tasks that require delicate manipulation, such as suturing, tissue dissection, and organ resection. Haptic feedback contributes to safer and more accurate surgeries and reduces the learning curve for surgeons transitioning to robotic techniques [53].

Sensory augmentation: Emerging technologies are pushing the boundaries of sensory augmentation in surgery. These innovations aim to provide surgeons with enhanced visual, auditory, or tactile information during surgery. For instance, augmented reality (AR) and virtual reality (VR) systems can overlay 3D visualizations, real-time data, or navigation guides onto a surgeon's field of view. This augmented sensory input can help surgeons more clearly visualize complex anatomical structures, critical landmarks, and instrument positioning. Additionally, sensory augmentation may include auditory cues or feedback that provide real-time information about tissue characteristics or instrument status. These advancements empower surgeons with a comprehensive understanding of the surgical site, leading to more informed decision-making and precise execution of procedures [54].

Personalized Surgery and Genomic Medicine

Genomic surgery: Integrating a patient's genomic data into surgical planning represents a groundbreaking advancement in personalized surgery. Genomic information can provide valuable insights into an individual's genetic makeup and susceptibility to specific diseases. Surgeons can leverage this data to tailor surgical procedures to the patient's unique genetic profile. For example, genetic markers may indicate a predisposition to certain complications or suggest the most effective treatment strategies. This personalized approach enables surgeons to optimize surgical interventions, improve outcomes, and minimize risks. By aligning surgical techniques with a patient's genetic characteristics, healthcare providers can offer more effective treatments and less likely to result in adverse events [55].

Three-dimensional printing: 3D printing technology has become an invaluable tool in personalized surgery. It allows for the creation of patient-specific surgical models and instruments, enabling surgeons to practice and plan procedures with unprecedented accuracy. These 3D-printed models replicate a patient's unique anatomy, giving surgeons a tangible and precise representation of the surgical site. Surgeons can rehearse complex procedures, assess potential challenges, and strategize their approach before surgery. This preoperative planning enhances surgical precision and reduces the risk of complications. Moreover, 3D printing enables the fabrication of customized surgical instruments tailored to the patient's anatomy, further enhancing the safety and efficacy of surgical interventions [56].

Regulatory and Ethical Considerations

Regulatory frameworks: As robotic surgery advances, regulatory bodies are actively developing guidelines and standards to ensure patient safety and the effectiveness of these technologies. Regulatory frameworks aim to establish precise requirements for robotic surgical systems' design, manufacturing, and operation. They may encompass equipment certification, surgeon training, maintenance protocols, and patient consent processes. These regulations are essential to mitigate risks and provide a framework for accountability, helping to ensure that robotic surgery adheres to the highest standards of safety and quality [57].

Ethical challenges: Integrating AI and automation in surgery raises many ethical challenges that warrant careful consideration. These challenges include liability in autonomous surgical procedures, privacy concerns regarding patient data and informed consent, and the role of human oversight in robotic surgery. Ethical guidelines must address issues such as ensuring transparency in AI-driven decision-making, safeguarding patient privacy, and defining the boundaries of autonomous surgical actions. Striking the right balance between the advantages of automation and the ethical principles that underpin medical practice is crucial to fostering public trust and ensuring the responsible use of robotic surgery [58].

Challenges and limitations

Cost and Accessibility

High initial costs: Robotic surgical systems' acquisition and maintenance costs can be substantial. Purchasing equipment and ongoing maintenance, training, and software updates require a significant financial investment. This financial barrier can be incredibly challenging for smaller hospitals and healthcare facilities with limited budgets. To address this, healthcare institutions may explore options such as group purchasing agreements, financing arrangements, or partnerships with more extensive facilities to make robotic surgery more accessible [59].

Economic disparities: The high cost of robotic surgery can exacerbate healthcare disparities, limiting access to advanced surgical options for patients in underserved or economically disadvantaged regions. Patients in such areas may need help receiving care from facilities that offer robotic surgery, leading to unequal access to the benefits of this technology. Healthcare providers and policymakers must work together to address these disparities by implementing strategies that ensure equitable access to robotic surgery, such as telemedicine programs or mobile surgical units [60].

Financial sustainability: Healthcare institutions must carefully evaluate the financial sustainability of robotic surgery programs. While robotic systems offer numerous benefits, including shorter hospital stays and reduced complications, the financial considerations are complex. Hospitals must balance the upfront costs with the potential long-term benefits and patient demand for robotic procedures. A thorough cost-benefit analysis should inform decisions about adopting and expanding robotic surgery programs, considering patient volume, reimbursement rates, and overall financial viability [61].

Learning Curve for Surgeons

Training requirements: Becoming proficient in robotic surgery demands specialized training and a significant commitment of time and effort. Surgeons must acquire the skills to operate robotic systems effectively, navigate the surgical console, and precisely manipulate robotic instruments. Training programs typically include hands-on simulations, supervised training on live patients, and proficiency assessments. These programs ensure surgeons are well prepared to safely handle robotic surgery's unique features and challenges. Continuous education and ongoing training are crucial to keep surgeons up-to-date with evolving technology and techniques [62].

Transition from conventional techniques: Experienced surgeons transitioning to robotic surgery may encounter distinct challenges as they adapt their skills and techniques to this technology. The transition from traditional open or laparoscopic surgery to robotic surgery involves a shift in surgical approach and the need to master a new set of tools. During this learning phase, patient outcomes may be affected as surgeons familiarize themselves with the nuances of robotic procedures. Healthcare institutions must provide support and resources to facilitate this transition, including mentorship, proctoring, and opportunities for skill refinement. Patient safety must be prioritized during this learning curve, and patients should be informed about the surgeon's experience with robotic surgery [63].

Technical Issues and Safety Concerns

Technical failures: Like any technology, robotic surgical systems can experience technical failures, from hardware malfunctions to software glitches. These failures may lead to interruptions or complications during surgery. Healthcare facilities must implement rigorous maintenance protocols and redundancy measures to mitigate these risks. Regular equipment inspections, software updates, and backup systems are essential to minimize the impact of technical failures on patient safety [64].

Intraoperative challenges: Surgeons operating with robotic systems must be prepared to handle unexpected intraoperative challenges. These may include robotic arm malfunctions, communication issues between the surgeon and the console, or unforeseen obstacles during the procedure. Surgeons and their surgical teams must receive training in troubleshooting and have contingency plans to ensure patient safety and the successful completion of the surgery [65].

Infection risk: Robotic systems introduce new infection risks if not adequately sterilized between procedures. Proper cleaning and sterilization protocols must be established and strictly adhered to, following the manufacturer's guidelines and best practices. Maintaining a sterile surgical environment is essential to prevent surgical site infections and other complications associated with microbial contamination [66].

Lack of haptic feedback: While haptic feedback improves in robotic surgical systems, they still lack the full range of tactile sensations available in traditional surgery. This limitation can affect a surgeon's ability to assess tissue properties, such as texture, tension, and elasticity. Surgeons must rely on visual and auditory cues and their experience and training to compensate for the absence of complete haptic feedback. Research and development efforts continue to work toward enhancing haptic feedback further to improve the safety and effectiveness of robotic surgery [67].

Ethical and Legal Challenges

Informed consent: Informed consent is a fundamental ethical principle in health care. Patients must comprehensively understand the risks and benefits of robotic surgery, including potential complications from using this technology. Additionally, patients should be informed about the surgeon's level of experience and training with robotic systems. It is incumbent upon healthcare providers to ensure that patients are fully informed, allowing them to make autonomous decisions about their treatment options. Adequate patient education materials and a straightforward, transparent consent process are essential to meeting this ethical obligation [68].

Liability issues: Introducing AI and autonomous features in robotic surgery raises complex questions regarding liability in case of errors or malfunctions. Determining responsibility when human surgeons collaborate with AI-driven robotic systems presents legal challenges. Healthcare institutions and regulatory bodies must work together to establish clear guidelines and frameworks for defining liability, particularly in cases where autonomous AI plays a significant role in surgical decision-making and execution. Addressing these legal complexities is essential to safeguarding both patient rights and the interests of healthcare professionals [69].

Privacy and data security: The collection and transmission of patient data during telesurgery or remote surgery raise significant concerns about privacy and data security. Particularly in cases involving sensitive medical information, maintaining the confidentiality and security of patient data is paramount. Ethical standards and legal regulations must be established and rigorously followed to safeguard patient data throughout the surgical process. This includes secure data transmission, storage, access controls, and comprehensive policies for data breach prevention and reporting [70].

Patient Acceptance

Limited awareness: A significant barrier to patient acceptance of robotic surgery is the limited awareness of this advanced technology. Many patients may need to be fully informed about robotic surgery or its advantages, leading to a lack of confidence in choosing this option when appropriate. To address this, healthcare providers must prioritize patient education and awareness campaigns. Patients can make more informed decisions about their healthcare by providing comprehensive information about robotic surgery, its benefits, and its suitability for specific conditions [71].

Perceived risk: Patients may have concerns about the safety and effectiveness of robotic surgery, which can deter them from considering this approach. Healthcare providers must communicate openly and transparently with patients to address their concerns and misconceptions. Sharing success stories and clinical outcomes data and emphasizing surgical teams' rigorous training and expertise using robotic systems can help alleviate perceived risks and build patient confidence [72].

Access to information: Access to accurate and unbiased information about robotic surgery can vary among patients, leading to disparities in knowledge and decision-making. Healthcare institutions should prioritize providing accessible and patient-friendly resources that explain robotic surgery clearly and understandably. Additionally, ensuring that patients can access reputable online and offline sources can empower them to make well-informed choices about their healthcare options [17].

Future outlook and implications

The Role of Robotic Surgery in Health Care

Mainstream integration: Robotic surgery is on the cusp of widespread integration into mainstream healthcare. It will offer an expanded array of minimally invasive options across various medical specialties. Robotic systems will cater to increasing surgical procedures as they become more versatile and adaptable. Patients can benefit from reduced trauma, quicker recoveries, and smaller incisions. Surgeons can access precise tools that enhance their capabilities, improving surgical outcomes [73].

Patient-centered care: The patient experience is poised for a transformation with the incorporation of robotic surgery into healthcare. Personalized treatment plans will take center stage, with genomic data and patient-specific information guiding surgical approaches. Surgeons can tailor procedures to individual patient profiles, optimizing outcomes and minimizing risks. This patient-centric model ensures that health care is not just about treating diseases but also about delivering care uniquely attuned to each patient's needs and genetic makeup [74].

Global healthcare access: Robotic surgery can potentially bridge healthcare gaps globally. Telesurgery and remote surgery are becoming increasingly feasible, enabling patients in remote and underserved areas to access specialized surgical expertise. This technology can facilitate real-time consultations and surgeries conducted by experts far from the patient. By reducing geographical barriers and enhancing access to surgical care, robotic surgery can reduce healthcare disparities and ensure that patients worldwide receive the best possible treatment, regardless of location [49].

Potential Impact on Healthcare Delivery

Efficiency and cost-efficiency: Further elaboration is required on the impact of robotic surgery on the overall surgery cost. While the current text highlights the potential efficiency gains through advancements in robotic technology and AI integration, it is important to delve deeper into how these improvements translate into specific cost reductions. Specifically, more detailed insights into how the precision and automation of robotic systems contribute to shorter surgery times, reduced blood loss, and faster recovery periods must be provided. The connection between these factors and the subsequent impact on hospital stays, postoperative care expenses, and the need for additional interventions must be clearly articulated. Additionally, it must be considered to discuss how hospitals and healthcare systems can optimize resources, ultimately leading to a potential reduction in the financial burden on both institutions and patients [75].

Outpatient and same-day surgery: As robotic systems become more sophisticated and minimally invasive, surgical procedures may transition to outpatient or same-day surgery settings. Patients can undergo surgeries and return home on the same day, eliminating the need for prolonged hospital stays. This shift can alleviate the demand for hospital resources, freeing up beds for more critical cases and enhancing overall healthcare system capacity. Additionally, outpatient and same-day surgery options offer patients greater convenience and comfort during their recovery, leading to a more patient-centered approach to healthcare delivery [41].

Preventive and early intervention: The precision and capabilities of robotic surgery extend beyond traditional treatment modalities. Robotic systems can facilitate preventive and early intervention strategies. For instance, the high-resolution imaging and enhanced dexterity of robotic instruments can enable the earlier detection and removal of precancerous or abnormal tissue. This proactive approach to health care can prevent disease progression, reduce the need for aggressive treatments, and improve long-term patient outcomes. By shifting the focus from late-stage interventions to early, less-invasive procedures, robotic surgery can contribute to a more holistic and preventive approach to health care [76].

Collaboration Between Robots and Surgeons

Integrating robotic technology in surgery has undoubtedly advanced the field, offering precision and efficiency that can enhance surgical outcomes. However, it is unlikely that surgeons will solely rely on robots in the foreseeable future. Surgeons possess a unique combination of adaptability, creativity, and intricate manual skills that current robotic systems may not fully replicate. Surgical procedures often demand on-the-spot decision-making and adjustments based on the patient's specific condition, aspects where human expertise excels. While robots provide stability and precision, their lack of sensory feedback, haptic perception, and the complexity of certain procedures present limitations. Moreover, the high initial costs, ongoing maintenance expenses, and the need for specialized training contribute to the challenges associated with widespread adoption. There is also a concern about overreliance on robotic assistance, potentially leading to an erosion of surgical skills. Striking a balance and ensuring that surgeons continue to develop and maintain their manual skills is crucial. Moreover, addressing patient and public perceptions about robotic surgery is essential for widespread acceptance. In essence, the collaboration between surgeons and robotic systems should be viewed as a synergistic relationship, with surgeons at the forefront, leveraging technology to augment their capabilities rather than replacing them entirely.

Human-robot synergy: The future of surgical collaboration will be marked by a dynamic synergy between human surgeons and robotic systems. Robots will assume routine and repetitive tasks, freeing surgeons to concentrate on the complex decision-making and critical elements of surgery that demand their expertise. This collaboration enhances the precision and efficiency of procedures while reducing the physical strain on surgeons. Robots will act as reliable extensions of the surgical team, amplifying human capabilities and ensuring consistent, high-quality outcomes [17].

Robot-assisted training: Robotic systems are set to play an increasingly pivotal role in surgeon training and education. These systems will provide aspiring surgeons with immersive and realistic simulations, replicating various surgical scenarios. Surgeon trainees will gain valuable hands-on experience and receive guidance from robotic systems, helping them refine their skills and build confidence. Additionally, experienced surgeons can use robotic platforms to hone their abilities, explore new techniques, and stay updated with the latest advancements in surgical practice. Ultimately, this training will contribute to a more skilled and proficient surgical workforce [77].

Remote assistance: The advent of remote surgical assistance will revolutionize healthcare delivery. Surgeons can access real-time support and guidance from robotic systems, experts, and AI algorithms, regardless of geographic location. This remote assistance will enable rapid consultations and collaborations between surgeons, fostering knowledge exchange and enhancing decision-making during complex procedures. Surgeons in remote or underserved areas can seek expert guidance, reducing disparities in access to specialized surgical expertise. Moreover, remote assistance can facilitate telesurgery, allowing skilled surgeons to perform procedures on patients far from their physical location and bringing expertise to regions with limited access to surgical care [78].

Research and Development Directions

AI and ML: As robotic surgery advances, so does the integration of AI and ML. Further development in AI and ML algorithms will usher in an era of autonomous surgical procedures. These algorithms will be designed to analyze patient data in real time, offering precise recommendations to surgeons during surgery. For instance, AI-driven robotic systems may assist in identifying critical structures, optimizing incision locations, and adapting to unforeseen complications seamlessly. Predictive analytics will play a pivotal role, allowing for the anticipation of patient-specific responses and complications, leading to a higher level of personalized care and optimized surgical outcomes [47].

Nanorobotics: Nanorobotics, operating at the cellular and molecular levels, is an exciting frontier in surgical research and development. Continued exploration of nanorobots holds the potential to revolutionize disease diagnosis and management. These miniature robotic agents could be designed to navigate the human body, targeting and treating diseases at their source with unparalleled precision. For example, nanorobots might deliver medications directly to cancer cells, perform delicate repairs at the cellular level, or remove plaque from arteries, all while minimizing collateral damage to healthy tissues. Research in nanorobotics promises to open new frontiers in the early detection and treatment of diseases [79].

Biocompatible materials: The development of biocompatible materials is indispensable for the next generation of robotic surgical systems. Advancements in materials science are essential to ensure that robotic systems can interact with the human body safely and efficiently. Biocompatible materials will be crucial for constructing robotic components, such as surgical instruments and implants, seamlessly integrating with human tissues. These materials should not trigger immune responses, be resistant to degradation within the body, and maintain their structural integrity over time. Research in biocompatible materials will contribute to creating surgical robots capable of achieving optimal surgical outcomes while minimizing the risk of adverse reactions and complications [80].

Ethical and legal frameworks: Investigating the ownership of databases within the realm of big data raises crucial ethical and legal inquiries. Determining who possesses the ownership rights to the databases and the ethical and legal aspects related to the sharing and utilization of such data are vital considerations. From an ethical standpoint, issues such as informed consent, transparency in decision-making, and the allocation of liability for errors or malfunctions must be thoroughly examined. Additionally, establishing clear, comprehensive, and adaptable ethical and legal frameworks is imperative. These frameworks should not only address the current challenges associated with robotic surgery, AI, and data privacy but also anticipate and accommodate future developments. In doing so, we can ensure patient safety, safeguard patient rights, and delineate the roles and responsibilities of both human surgeons and autonomous robotic systems. This ongoing research and development effort is essential to responsibly and ethically navigate the evolving landscape of robotic surgery [81].

## Conclusions

In conclusion, robotic surgery is a testament to human innovation and the relentless pursuit of excellence in health care. This comprehensive overview has illuminated the field's evolution from its historical roots to its current prominence, underscoring its vital role in modern medicine. Robotic surgery has already profoundly impacted patient care with its precision, reduced complications, and faster recovery times. As we peer into the future, it becomes evident that this transformative field will continue to shape the healthcare landscape, offering the promise of more accessible, efficient, and personalized surgical interventions. While challenges persist, such as cost considerations and ethical complexities, they are eclipsed by the boundless potential for improving patients' lives worldwide. Robotic surgery's journey is far from over. As it advances, it reaffirms our commitment to advancing the frontiers of medical science and providing the highest standards of care to those in need.
